# Whole-liver radiotherapy for end-stage colorectal cancer patients with massive liver metastases and advanced hepatic dysfunction

**DOI:** 10.1186/1748-717X-5-97

**Published:** 2010-10-26

**Authors:** Seung-Gu Yeo, Dae Yong Kim, Tae Hyun Kim, Sun Young Kim, Yong Sang Hong, Kyung Hae Jung

**Affiliations:** 1 Center for Colorectal Cancer, Research Institute and Hospital, National Cancer Center, Goyang, Korea; 2 Department of Radiation Oncology, Soonchunhyang University College of Medicine, Cheonan, Korea; 3 Department of Oncology, Asan Medical Center, University of Ulsan College of Medicine, Seoul, Korea

## Abstract

**Background:**

To investigate whether whole-liver radiotherapy (RT) is beneficial in end-stage colorectal cancer with massive liver metastases and severe hepatic dysfunction.

**Methods:**

Between June 2004 and July 2008, 10 colorectal cancer patients, who exhibited a replacement of over three quarters of their normal liver by metastatic tumors and were of Child-Pugh class B or C in liver function with progressive disease after undergoing chemotherapy, underwent whole-liver RT. RT was administered using computed tomography-based three-dimensional planning and the median dose was 21 Gy (range, 21-30) in seven fractions. Improvement in liver function tests, defined as a decrease in the levels within 1 month after RT, symptom palliation, toxicity, and overall survival were analyzed retrospectively.

**Results:**

Levels of alkaline phosphatase, total bilirubin, aspartate transaminase, and alanine transaminase improved in 8, 6, 9, and all 10 patients, respectively, and the median reduction rates were 42%, 68%, 50%, and 57%, respectively. Serum carcinoembryonic antigen level decreased after RT in three of four assessable patients. For all patients, pain levels decreased and acute toxicity consisted of nausea/vomiting of grade ≤ 2. Further chemotherapy became possible in four of 10 patients. Mean survival after RT was 80 ± 80 days (range, 20-289); mean survival for four patients who received post-RT chemotherapy was 143 ± 100 days (range, 65-289), versus 38 ± 16 days (range, 20-64) for the six patients who did not receive post-RT chemotherapy (*p *= 0.127).

**Conclusions:**

Although limited by small case number, this study demonstrated a possible role of whole-liver RT in improving hepatic dysfunction and delaying mortality from hepatic failure for end-stage colorectal cancer patients with massive liver metastases. Further studies should be followed to confirm these findings.

## Background

Most colorectal cancer deaths are attributable to distant metastases, frequently in the liver. At diagnosis, approximately 20% of colorectal cancer patients have liver metastases, and about half of patients initially diagnosed with localized disease develop metachronous liver metastases [1-3]. Curative resection of liver metastasis is possible in fewer than 25% of patients and two-thirds of patients receiving liver resections show liver disease recurrence within 2 years [4-7]. Systemic and hepatic arterial chemotherapy are the principal treatments for unresectable or recurrent colorectal cancer liver metastasis [5,8]. Unfortunately, a subset of these patients progress to end-stage status, in which their livers are replaced by metastatic tumors. This eventually leads to hepatic failure and death [3,9]. For these patients, further chemotherapy cannot be given due to severe hepatic dysfunction or metastatic tumors which became refractory to chemotherapy.

Palliative whole-liver radiotherapy (RT) has been an effective treatment in symptom control for intra-hepatic tumors including colorectal cancer liver metastases [3,10]. Recent studies, using advanced RT technologies, have investigated the delivery of escalated radiation doses to partial liver volumes and administering concurrent chemotherapy to increase the effectiveness of RT on the tumor [5,11-15]. However, for end-stage colorectal cancer patients with massive liver metastases and advanced hepatic dysfunction, liver RT is not usually provided unless associated symptoms are severe and uncontrollable. If RT can improve advanced hepatic dysfunction, further chemotherapy treatment might become possible and patient mortality due to hepatic failure could be postponed.

The present study explored the efficacy of palliative whole-liver RT for end-stage colorectal cancer patients with massive liver metastases and impending hepatic failure on liver function improvement and patient survival.

## Methods

### Patients

The institutional review board of the National Cancer Center approved this study. Informed consent was not required because the study was retrospective in nature. Between June 2004 and July 2008, 10 end-stage colorectal cancer patients with massive liver metastases and advanced hepatic dysfunction who received whole-liver RT were retrospectively analyzed. Inclusion criteria were massive liver metastases defined as the replacement of over three-quarters of the liver by metastatic tumors, and advanced hepatic dysfunction defined as Child-Pugh class B or C. Patient characteristics are shown in Table 1. Nine patients were females and the median age was 57 years (range, 42-73). All patients were in the Eastern Cooperative Oncology Group performance scale range of 2-4. The principal symptoms prior to RT were mild-to-moderate abdominal pain and distension resulting from the growing liver metastases.

**Table 1:** Patient characteristics

Patient No.	Age (yr)	Gender	Primary tumor site	Initial stage	Surgical resection		Extrahepatic metastasis at initial diagnosis	Interval from liver metastasis to RT (mo)	Extrahepatic metastasis at the time of RT	Serum CEA* before RT (ng/mL)	Child-Pugh classification before RT
										
					Primary	Liver					
1	47	F	Rectum	pT3N0M1	Y	N	Y	30	Y	1727	B
2	57	M	Colon	pT3N0M1	Y	Y	Y	18	Y	1481	C
3	53	F	Colon	pT4N1M1	Y	N	Y	18	Y	11858	B
4	42	F	Rectum	pT3N2M1	Y	Y	Y	13	Y	5198	B
5	61	F	Colon	pT4N2M1	Y	N	N	4	N	7629	B
6	68	F	Colon	cT3N2M1	N	N	Y	6	Y	10^†^	C
7	45	F	Colon	pT3N2M1	Y	N	N	26	Y	2103	B
8	57	F	Colon	pT3N2M1	Y	N	Y	11	Y	373	B
9	70	F	Colon	pT3N0M1	Y	N	Y	11	Y	6424	B
10	73	F	Rectum	cT3N2M1	N	N	Y	8	Y	4092	B

*Carcinoembryonic antigen (normal range, ≤ 5 ng/mL)

^†^Measured 3 months before RT. Levels in other patients were assessed 1 month before RT

Primary tumor sites were the colon and rectum in seven and three patients, respectively. At the initial diagnosis of primary colorectal cancer, all patients exhibited liver metastases, and eight of them also had extra-hepatic metastases. Surgical resection for primary colorectal tumors was performed with curative intent in four (No. 1, 2, 4, 5) and palliative in four patients (No. 3, 7, 8, 9). Surgery was not performed on either the primary tumor or on the liver in the remaining two patients (No. 6, 10); because multiple organ metastases were found at initial diagnosis, chemotherapy alone was given. In two of the patients who received synchronous surgery for both primary tumors and liver metastasis, metastatic disease recurred in the liver at 13 (No. 2) and 3 months (No. 4). The median interval from initial liver metastasis diagnosis to liver RT was 12 months (range, 4-30). The median serum carcinoembryonic antigen (CEA) level measured 1 month prior to RT was 4092 ng/mL (range, 373-11858). Child-Pugh classification before RT was class B in eight patients and class C in the remaining two patients. All patients received multiple cycles (median, 13; range, 4-32) of chemotherapy before RT. This included various combinations of 5-fluorouracil, leucovorin, capecitabine, irinotecan, oxaliplatin, S-1, uracil-tegafur, bevacizumab, and cetuximab. Patients were referred for liver RT when they became refractory to chemotherapy or could not tolerate further chemotherapy due to hepatic dysfunction or medical comorbidities. At the time of liver RT, nine patients had extra-hepatic disease with metastases in the para-aortic lymph node, peritoneum, lung, pelvis, bone, or supraclavicular lymph nodes.

### Radiotherapy

**Figure 1:**
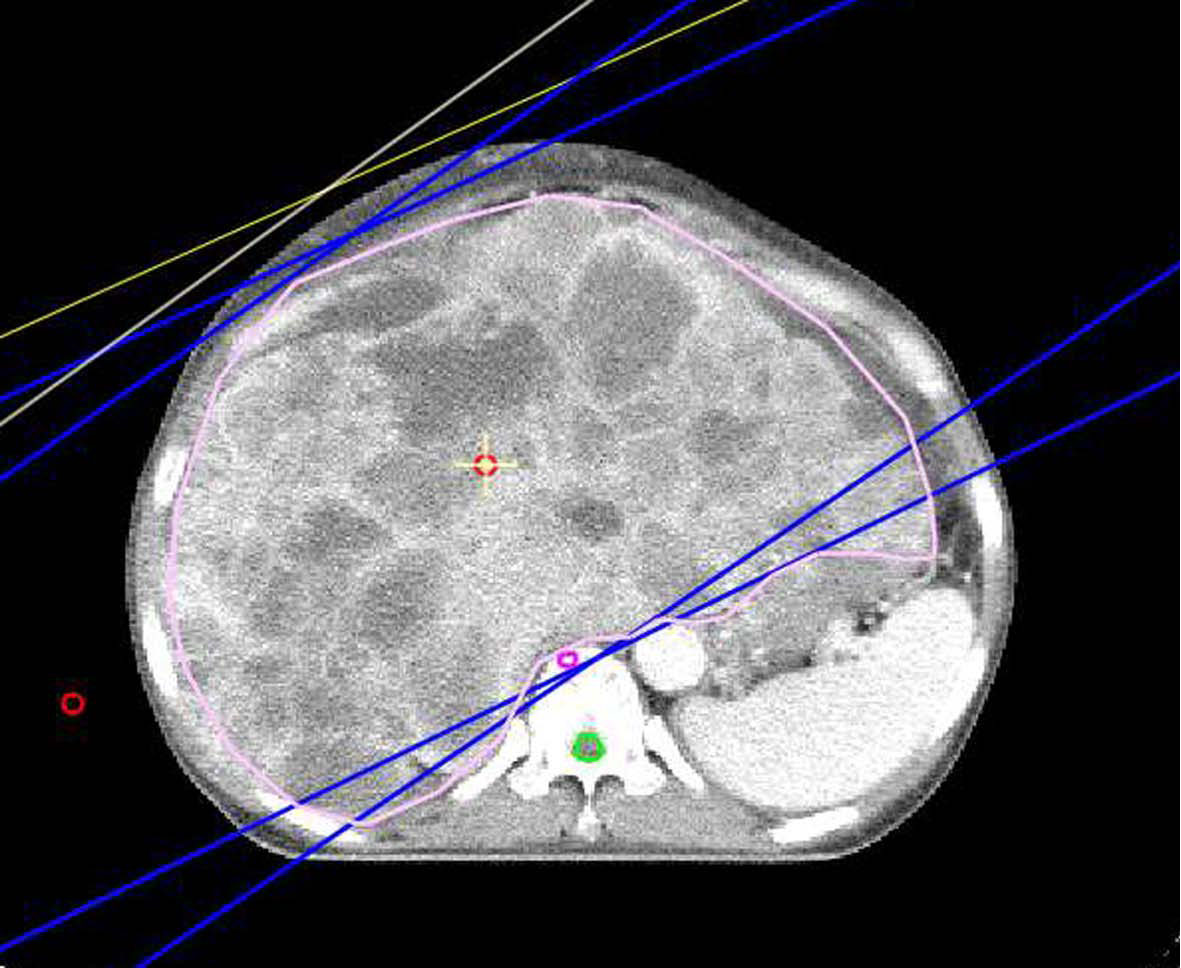
**Central axial computed tomography slice showing the three-dimensional conformal radiotherapy plan**. The treatment target was the whole-liver. Two radiation fields, with left anterior oblique and right posterior oblique beams, both consisting of 15 MV X-rays, were used to cover the target.

All patients were placed in the supine position with arms above their heads and immobilized using an alpha-cradle device to improve the setup reproducibility during the planning and treatments and to facilitate the use of lateral radiation ports. Computed tomography data were transferred to a three-dimensional conformal RT planning system (PROWESS, Alliant Medical Technology, Chico, CA). For each patient, clinical target volume including the entire liver metastatic tumors and neighboring organs, such as stomach, duodenum, kidneys and spinal cord, were contoured on each slice and reconstructed three-dimensionally. The planning target volume included almost the entire liver, without a margin, to minimize radiation damage to neighboring normal organs. All patients were asked to respire shallowly to minimize target motion. Two radiation portals were used: a left anterior oblique and a right posterior oblique beam (Figure 1). RT was administered via a linear accelerator that emits X-rays of 15 MV. A daily dose of 3 Gy was administered five times per week to deliver a total dose of 21 Gy; this scheme was determined based on the Radiation Therapy Oncology Group study for hepatocellular carcinoma [16]. Chemotherapy was not administered during RT, except that one patient, No. 7, received concurrent cetuximab.

### Analysis

To evaluate the effect of RT on liver function improvement, liver function tests, including alkaline phosphatase (ALP), total bilirubin (TB), aspartate transaminase (AST), alanine transaminase (ALT), and serum CEA levels before and after RT, were analyzed. Improvement in these values was defined as any decrease in the levels within 1 month after RT. The reduction rate was calculated between the level before RT and the lowest level recorded within 1 month after RT. Overall survival was calculated from the first day of RT to death and compared using the *t*-test. Treatment toxicity was evaluated using the Radiation Therapy Oncology Group radiation morbidity scoring criteria. Statistical tests were performed using the SPSS software (ver. 14.0; SPSS Inc., Chicago, IL, USA). A *p-*value of < 0.05 was deemed to indicate statistical significance.

## Results

Treatment results are shown in Table 2. Nine patients received 21 Gy in seven fractions and one patient received 30 Gy in 10 fractions. All patients experienced a decrease in pain levels, as measured using numeric rating scales. Treatment response based on abdominopelvic computed tomography was assessed in four patients: three (No. 2, 5, 7) showed minimal responses, while one (No. 1) had no response. Treatment-related acute toxicity consisted mostly of nausea/vomiting, which was grade 1 or 2.

**Table 2:** Treatment results

Patient No.	Dose (Gy)/fraction	Alkaline phosphatase (35 - 104 IU/L)*	Total bilirubin (0.2 - 1.2 mg/dL)*	Aspartate transaminase (0 - 40 IU/L)*	Alanine transaminase (0 - 40 IU/L)*	Post-RT chemo-therapy	Survival from RT (day)
1	21/7	^†^N (306)	Y (6.5 → 2.2, 66%)	Y (118 → 69, 42%)	Y (43 → 27, 37%)	Y	123
2	21/7	^‡^Y (1246 → 350, 72%)	Y (3.0 → 1.6, 47%)	Y (114 → 29, 75%)	Y (43 → 7, 84%)	Y	94
3	21/7	N (554)	N (1.8)	Y (152 → 109, 28%)	Y (30 → 18, 40%)	N	64
4	21/7	Y (432 → 297, 31%)	Y (1.7 → 1.2, 29%)	Y (60 → 24, 60%)	Y (35 → 13, 63%)	N	22
5	21/7	Y (415 → 89, 79%)	Y (3.2 → 1.0, 69%)	Y (197 → 20, 90%)	Y (80 → 13, 84%)	Y	289
6	21/7	Y (675 → 366, 46%)	Y (15.8 → 4.2, 73%)	Y (101 → 33, 67%)	Y (49 → 23, 53%)	N	44
7	30/10	Y (1093 → 613, 44%)	Y (36.7 → 9.2, 75%)	Y (250 → 124, 50%)	Y (123 → 31, 75%)	Y	65
8	21/7	Y (615 → 376, 39%)	N (1.3)	Y (88 → 69, 22%)	Y (25 → 15, 40%)	N	34
9	21/7	Y (404 → 348, 14%)	N (7.0)	N (42)	Y (31 → 19, 39%)	N	44
10	21/7	Y (612 → 500, 18%)	N (8.4)	Y (142 → 107, 25%)	Y (76 → 30, 61%)	N	20

*Normal range

^†^No improvement in liver function test. Level before RT in parentheses

^‡^Improvement in liver function test. Levels before and after RT, and the reduction rate in parentheses

The levels of ALP, TB, and AST were above normal prior to RT in all patients (median 583 IU/L (range, 306-1246), median 6.5 mg/dL (range, 1.3-36.7), and median 118 IU/L (range, 42-250), respectively). After RT, ALP, TB, and AST levels decreased in eight (80%), six (60%), and nine (90%) patients, respectively, and the median reduction rates were 42% (range, 14-79%), 68% (range, 29-75%), and 50% (range, 22-90%), respectively. ALT level was higher than normal prior to RT in eight patients (median, 43 IU/L; range, 25-123). After RT, ALT level improved in all patients and the median reduction rate was 57% (range, 37-84%). Serum CEA levels 1 month after RT were available for four patients; these levels decreased by 223-779 ng/mL in three patients (No. 1, 5, 7) and increased by 271 ng/mL in one patient (No. 2). The serum albumin (g/dL) change was small (within ± 15%) in all patients, and four of five patients with prothrombin time (international normalized ratio) specified within 1 month after RT showed a decrease (10-25%) in that value. Child-Pugh score was specified in five patients within 1 month after RT; it decreased (from -1 to -3) in three and was unchanged in two. The duration of liver function palliation varied according to the different measures of liver function tests, and the improved liver functions deteriorated before the patients died. As an example, the total bilirubin level improvement lasted for 1 (No. 4, 6), 2 (No. 1, 7), 3 (No. 2), or 7 months (No. 5) in six patients with post-RT improvement in this value.

Four patients received post-RT chemotherapy, which was decided by medical oncologists based on the performance status of the patients and the availability of effective agents: seven cycles of bevacizumab (No. 1), nine cycles of 5-fluorouracil, leucovorin, and oxaliplatin (No. 2), 19 cycles of cetuximab and nine cycles of irinotecan (No. 5), or four cycles of cetuximab (No. 7). For all patients, the mean survival times from the initial surgery or diagnosis of primary colorectal tumor and from RT were 19 ± 11 months (range, 7-42) and 80 ± 80 days (range, 20-289), respectively. The mean survival times from RT in the four patients who received post-RT chemotherapy and the remaining six patients who did not were 143 ± 100 days (range, 65-289) and 38 ± 16 days (range, 20-64), respectively (*p *= 0.127).

## Discussion

Medical interventions that can possibly affect patient life span are often an issue in medial multidisciplinary decision-making for end-stage cancer patients who have only a few remaining days of life [17,18]. Patients in this study had end-stage colorectal cancer with multiple organ metastases, including massive liver metastases refractory to multiple chemotherapeutic drugs and liver function had progressed to almost hepatic failure. Performance status was unfavorable. Although pain associated with massive liver metastases was not severe, because of analgesic management and supportive care, we provided short-course palliative whole-liver RT and the advanced liver dysfunction was improved in most of the patients. Moreover, four patients were able to receive further chemotherapy due to improvement in liver function post-RT and we experienced delayed mortality in these end-stage colorectal cancer patients with massive liver metastases.

When survival time is estimated to be weeks-to-months, estimating life expectancy is difficult [19]. Kemeny et al. [9] compared hepatic arterial infusion versus systemic therapy for colorectal cancer patients with liver metastasis, and the independent prognostic factors for survival included percent liver involvement, synchronous disease, performance status, number of liver lesions, baseline CEA, and the levels of ALP and albumin. Wang *et al. *[20] analyzed predictors of survival after hepatic resection in colorectal cancer patients with liver metastasis, and they reported age, primary tumor grade, comorbidity score, and chemotherapy after liver resection to be significant prognostic factors. For patients in our study who exhibited several of these poor prognostic factors, their life expectancy was estimated to be less than several weeks. Usually, only supportive care is provided for such patients.

Several previous studies have investigated the effectiveness of whole-liver RT for the management of patients with liver metastases. Most of the patients included in these studies had liver metastases from colorectal primaries following chemotherapy and were treated with a total dose of 20-30 Gy at 1.5-3.0 Gy per fraction. Pain relief was achieved in 50-90% of the patients and up to 50% experienced a decrease in liver size, with an improvement in liver function tests [3,10]. More recent research has been directed at methods to amplify the effects of radiation on the tumor (hypoxic cell sensitizers and chemotherapy) and to deliver radiation more selectively to the target, while sparing the surrounding normal liver [5,11-15]. Higher doses of radiation could safely be applied to a hepatic lesion if functional liver tissue is spared from the high prescription dose. However, both the disease and patient status in these studies were more favorable than those of the current study. In a study by Mohiuddin *et al. *[11], which reported improvement in symptoms and survival with an additional boost dose to the dominant disease in colorectal cancer multiple liver metastases, Karnofsky performance score ≥ 80 was in 75.6% (34/45) patients and more than half of the patients had normal level of CEA and liver function tests. Krishnan *et al. *[5] reported favorable survival in colorectal cancer multiple liver metastases with increased RT dose targeting only the dominant tumor. All patients had Karnofsky performance scores ≥ 70 and the median number of hepatic tumors was four (range, 1-10). Whether whole-liver RT could be beneficial even for patients like those in the present study, who had disseminated liver metastases and in whom the hepatic functional reserve was totally exhausted, has not been previously assessed.

Systemic chemotherapy is the mainstay of treatment for massive metastatic colorectal cancer. Currently, regimens incorporating irinotecan, oxaliplatin, cetuximab, bevacizumab, and fluoropyrimidine are used for colorectal cancer liver metastasis. With the introduction of these new agents, the median survival time has increased to 15-21 months with first-line therapy and 7-12 months with second-line therapy [5,8,21]. Our patients had liver metastasis at initial diagnosis. They received multiple cycles of chemotherapy with or without liver metastasis resection, but metastatic disease progressed in the livers that became refractory to chemotherapy. Locoregional approaches, such as hepatic artery infusion chemotherapy, chemoembolization, cryotherapy, radiofrequency ablation, laser, and microwave coagulation, have shown promising results in these situations [3,5]. However, these options are limited by tumor size, proximity to vascular or biliary structures and the ability of patients to tolerate invasive procedures. RT does not have these limitations and is applicable to end-stage massive liver metastases with advanced hepatic dysfunction. Its efficacy was shown by an improvement in most of the liver function tests in all patients and a decrease in serum CEA levels for three of four assessable patients with acceptable morbidity. Because of improvement in liver function, four patients could receive additional chemotherapy after liver RT. Although statistical significance was not shown, due to the low statistical power of small sample size, the median survival time of patients who received chemotherapy after RT was longer than those of patients who did not.

## Conclusions

Although conclusions must be drawn carefully from a single-institution experience with a small sample size, our data suggest that short-course whole-liver RT can improve symptoms and hepatic dysfunction in end-stage colorectal cancer patients with massive liver metastases and severe hepatic dysfunction, and subsequently prolong their survival with acceptable morbidity. Further careful and larger scaled studies should be conducted to clarify the efficacy of whole-liver RT for these end-stage colorectal cancer patients with massive liver metastases.

## Competing interests

The authors declare that they have no competing interests.

## Authors' contributions

DYK contributed to conception and design of the study, and revised the manuscript. SGY contributed to analysis and interpretation of data, and drafted the manuscript. THK & YSH participated in revising the manuscript. SYK participated in data acquisition and literature research. KHJ contributed to conception of the study. All authors read and approved the final manuscript.
